# MRI-Based Texture Analysis of the Extraocular Optic Nerve in Idiopathic Parkinson’s Disease

**DOI:** 10.3390/jcm15145388

**Published:** 2026-07-09

**Authors:** Seda Nida Karakucuk, Murat Baykara, Cemile Buket Tugan Yıldız

**Affiliations:** 1 Department of Radiology, Faculty of Medicine, Kahramanmaras Sutcu Imam University, Kahramanmaraş 46040, Turkey; 2 Department of Radiology, Haydarpasa Numune Training and Research Hospital, University of Health Sciences, Istanbul 34752, Turkey; muratbaykara@hotmail.com; 3 Department of Neurology, Faculty of Medicine, Kahramanmaras Sutcu Imam University, Kahramanmaraş 46040, Turkey; bukettugan@yahoo.com

**Keywords:** Parkinson’s disease, optic nerve, magnetic resonance imaging, texture analysis, radiomics

## Abstract

**Objective**: To evaluate microstructural and morphological alterations of the extraocular optic nerve in patients with idiopathic Parkinson’s disease (IPD) using MRI-based texture analysis and to compare these findings with those of healthy controls. **Methods**: This retrospective study included 74 participants (37 IPD patients and 37 age- and sex-matched controls). IPD diagnosis was established according to the UK Parkinson’s Disease Society Brain Bank criteria. Coronal T2-weighted images obtained from a 1.5-T MRI system were retrospectively analyzed. A round region of interest was placed on the intraorbital extraocular segment of the optic nerve for histogram-based and radiomic texture analyses. Optic nerve sheath diameter (ONSD) was also measured. As no significant differences were observed between right and left eyes, the mean value of both eyes was used for statistical analyses. Statistical analyses included independent samples *t*-test, Mann–Whitney U test, and chi-square test. **Results**: No significant differences were observed between groups regarding age or sex (*p* > 0.05). ONSD was significantly greater in the IPD group than in healthy controls (5.2 ± 0.8 mm vs. 3.2 ± 0.5 mm, *p* < 0.05). Histogram analysis demonstrated significantly lower entropy values in patients with IPD (*p* < 0.05). In GLRLM analysis, short-run emphasis and high gray-level run emphasis were significantly lower, whereas long-run emphasis and low gray-level run emphasis were significantly higher in the IPD group (*p* < 0.05). GLSZM analysis revealed increased small zone emphasis and decreased large zone emphasis parameters in patients with IPD compared with controls (*p* < 0.05). **Conclusions**: MRI-based texture analysis reveals significant structural and microstructural alterations in the extraocular optic nerve in IPD, supporting its potential role as a non-invasive imaging biomarker for subclinical visual pathway involvement.

## 1. Introduction

Idiopathic Parkinson’s disease (IPD) is a progressive neurodegenerative disorder primarily characterized by the degeneration of dopaminergic neurons in the substantia nigra pars compacta [1]. While its hallmark features are motor-related, growing evidence indicates that dopaminergic depletion is not restricted to the basal ganglia but also extends to the retina, particularly affecting retinal ganglion cells [2]. This reduction in retinal dopamine may contribute to visual dysfunction through alterations in retinal signaling and visual pathway processing. Visual dysfunction is a common yet often underrecognized manifestation of Parkinson’s disease. In this context, intraocular structures have been extensively investigated using optical coherence tomography (OCT). Several studies have demonstrated that thinning of the retinal nerve fiber layer is associated with disease severity in IPD [3,4,5]. These findings suggest that the neurodegenerative process in Parkinson’s disease may involve not only central motor pathways but also the visual system. However, despite these insights, the extent of optic nerve involvement remains unclear, and the available literature addressing this issue is limited.

In recent years, quantitative imaging approaches have gained increasing importance in the evaluation of subtle tissue alterations. Texture analysis, in particular, enables the assessment of pixel intensity, spatial distribution, and gray-level variations within medical images [6]. This technique has been widely utilized in radiological practice across various imaging modalities. Notably, MRI-based texture analysis has demonstrated the ability to detect microstructural changes associated with acute inflammation and neurodegeneration, even when conventional imaging findings appear normal [7,8].

Structural and microstructural changes in the optic nerve and visual pathways have been reported in conditions such as Alzheimer’s disease, atypical Parkinsonian syndromes, and amyotrophic lateral sclerosis, likely driven by shared mechanisms like abnormal protein aggregation and neuroaxonal degeneration [9,10,11]. Therefore, evaluating optic nerve involvement in IPD within this broader neurodegenerative framework may help to clarify whether similar pathological processes affect the visual pathway. Studies in patients with optic neuritis have shown that MRI-based texture analysis can reveal subclinical optic nerve involvement, even when conventional MRI findings appear normal. Furthermore, this method has been proposed as a useful tool for both diagnosis and monitoring treatment-related recovery [12,13]. These findings highlight the potential of texture analysis as a sensitive imaging biomarker for detecting early or subtle tissue changes. Despite these advances, the application of MRI-based texture analysis to evaluate optic nerve involvement in IPD has not been sufficiently investigated. This gap is particularly important, as conventional imaging techniques may be inadequate for identifying early-stage or subclinical neurodegenerative changes in the visual pathway.

Accordingly, the present study aimed to characterize extraocular optic nerve involvement in idiopathic Parkinson’s disease using MRI-based texture analysis and optic nerve sheath diameter measurements. By combining quantitative imaging biomarkers with morphologic assessment, we sought to identify subtle optic nerve alterations that may not be detectable on conventional imaging, thereby contributing to the understanding of optic nerve involvement in IPD and expanding the current literature.

## 2. Materials and Methods

### 2.1. Study Design and Patient Characteristics

This retrospective study was conducted following approval from the Ethics Committee of Kahramanmaras Sutcu Imam University Faculty of Medicine (approval number: 2025/05). The study adhered to the principles of the Declaration of Helsinki (2000). Due to its retrospective design and the use of anonymized imaging data, the requirement for informed consent was waived.

A total of 74 participants were included in the study, comprising 37 patients diagnosed with IPD and 37 age-matched healthy controls. For the IPD group, the diagnosis was established by an experienced neurologist based on the UK Parkinson’s Disease Society Brain Bank clinical diagnostic criteria. Both eyes of each participant were initially evaluated (74 eyes in the IPD group and 74 eyes in the healthy control group). As no statistically significant differences were observed between right and left eye measurements, the mean value of both eyes was calculated for each participant and used as a single subject-level parameter for all subsequent statistical analyses (Supplementary Material).

The study population consisted of 52 males (70.3%) and 22 females (29.7%), with an age range of 36 to 86 years.

Patients were included based on the availability of brain MRI scans in the hospital’s Picture Archiving and Communication System (PACS). Control subjects were selected based on the absence of any known neurological or neurodegenerative disorders.

To ensure the reliability of texture analysis, the following exclusion criteria were applied: history of optic nerve disorders (e.g., optic neuritis, glaucoma, or optic atrophy), prior ocular surgery or significant orbital trauma, presence of other neurodegenerative diseases (e.g., multiple sclerosis or Alzheimer’s disease), hereditary optic neuropathies, neuromuscular disorders affecting the visual pathways, genetic forms of parkinsonism, and documented exposure to medications known to induce toxic optic neuropathy (e.g., selected antibiotics, antineoplastic agents, or other neurotoxic drugs). In addition, subjects with inadequate MRI image quality due to motion artifacts or technical limitations preventing accurate Region of Interest (ROI) placement on the optic nerve were excluded from the analysis.

### 2.2. MRI Examination

Cranial MRI examinations were performed using a 1.5 Tesla MRI system with a dedicated head coil (Philips Ingenia 1.5T, Eindhoven, The Netherlands). Coronal T2-weighted images were obtained using the following parameters: repetition time (TR)/echo time (TE): 4833/100 ms, field of view (FOV): 220 × 183 mm, and matrix size: 356 × 209 mm. Images were acquired with a slice thickness of 5 mm and an interslice gap of 1 mm, yielding a total of 24 coronal sections.

### 2.3. ROI Determination and Texture Analysis

**Figure 1:**
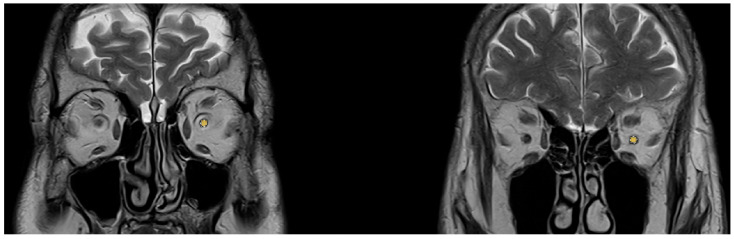
Coronal T2-weighted orbital MRI images used for optic nerve evaluation. The left panel represents a healthy control subject, while the right panel shows a patient with idiopathic Parkinson’s disease. ROI, shown in yellow, was placed on the intraorbital segment of the optic nerve for quantitative texture analysis.

Radiomics features were extracted from the intraorbital extraocular segment of the optic nerve. A single experienced radiologist, blinded to the clinical data, performed the manual segmentation using a dedicated workstation. A round-shaped 2D ROI was manually drawn on a single coronal T2-weighted image obtained at least 3 mm posterior to the globe. The ROI size was adjusted according to the diameter of the optic nerve (approximately 3–15 mm^2^) to include the optic nerve parenchyma while carefully excluding the surrounding optic nerve sheath and cerebrospinal fluid. This approach was adopted to ensure anatomical coverage of the optic nerve while minimizing contamination from adjacent structures. Care was taken to exclude adjacent orbital tissues from the ROI (Figure 1). The optic nerve sheath diameter (ONSD) measurements were performed at the same anatomical level and recorded in millimeters (mm). All ROI placements, ONSD measurements, and texture analyses were performed by the same radiologist.

ROI boundaries were manually drawn to encompass the optic nerve using a dedicated workstation (27-inch iMac, Apple Inc., Cupertino, CA, USA). Pixel data within each ROI were subsequently exported as XML (eXtensible Markup Language) files. Texture analysis evaluation of the obtained ROIs was performed on a Windows 10 (Microsoft Corporation, One Microsoft Way, Redmond, WA, USA) computer using an in-house MATLAB program (version R2021a; Math Works, Natick, MA, USA).

### 2.4. Statistical Analysis

Descriptive statistics were expressed as mean, standard deviation, median, minimum, and maximum values, as well as frequencies and percentages where appropriate. The normality of data distribution was assessed using the Kolmogorov–Smirnov and Shapiro–Wilk tests.

For comparisons of independent quantitative variables, the independent samples *t*-test was used for normally distributed data, while the Mann–Whitney *U* test was applied for non-normally distributed data. Categorical variables were compared using the Chi-square test. Receiver operating characteristic (ROC) curve analysis was performed to evaluate the diagnostic performance of ONSD and to determine the optimal cutoff value. The area under the curve (AUC), sensitivity, specificity, and 95% confidence intervals were calculated. Effect size for the primary outcome measure (ONSD) was calculated using Cohen’s d.

All statistical analyses were performed using SPSS software (version 28.0; IBM Corp., Armonk, NY, USA), and a *p*-value of <0.05 was considered statistically significant.

## 3. Results

### 3.1. Demographic and Clinical Characteristics

A total of 74 participants (37 patients with IPD and 37 healthy controls) were included in the study. Both eyes of all participants were initially evaluated (74 eyes in the IPD group and 74 eyes in the healthy control group). As no significant differences were observed between right and left eye measurements, the mean value of both eyes was calculated for each participant and used as the subject-level variable in all subsequent statistical analyses. The study population consisted of 22 females (29.7%) and 52 males (70.3%), with an age range of 36–86 years. The mean age was 63.5 ± 10.3 years in the IPD group and 62.3 ± 12.3 years in the control group. There were no statistically significant differences between the IPD and control groups in terms of age or sex distribution (*p* > 0.05).

The difference in ONSD between the IPD and control groups corresponded to a very large effect size (Cohen’s d = 3.00).

ONSD was significantly higher in the IPD group (5.2 ± 0.8 mm) compared to the control group (3.2 ± 0.5 mm) (*p* < 0.05).

**Figure 2:**
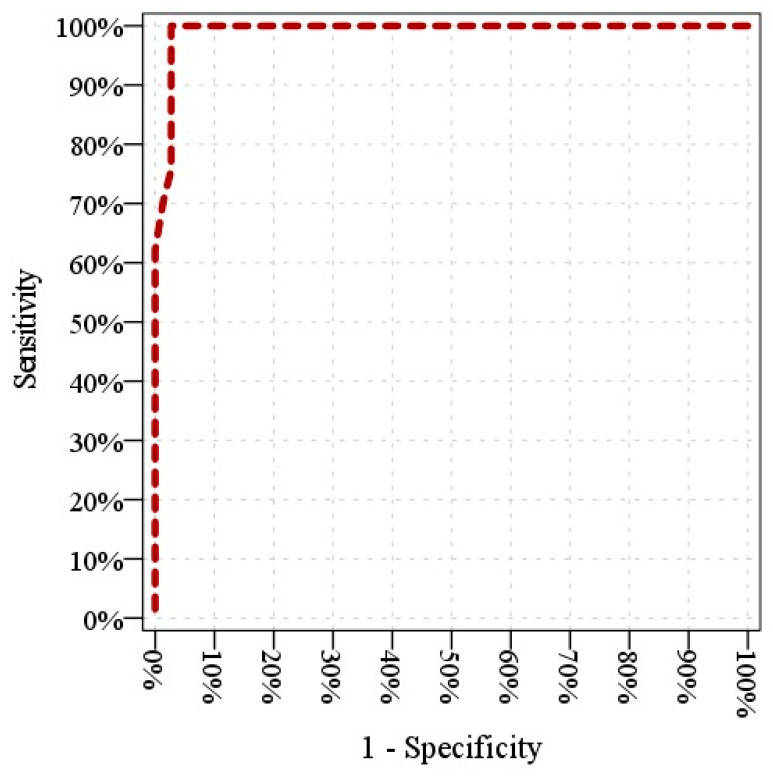
Receiver operating characteristic (ROC) curve of optic nerve sheath diameter (ONSD) for differentiating patients with idiopathic Parkinson’s disease (IPD) from healthy controls. The red dashed line represents the ROC curve of ONSD.

ONSD demonstrated a significant discriminative performance in differentiating between case and control groups, with an area under the curve (AUC) of 0.992 (95% CI: 0.980–1.000) (Figure 2).

### 3.2. Histogram-Based Texture Analysis

Compared to controls, the IPD group demonstrated significantly lower values for Pixel Count of ROI and Area of ROI (*p* < 0.05).

Histogram-derived parameters revealed that the IPD group had significantly higher mean, median, minimum, most frequent value, and root-mean-square values (*p* < 0.05 for all). Additionally, multiple lower and mid-range percentile values (1st, 3rd, 5th, 10th, 25th, 75th, and 90th percentiles) were significantly elevated in the IPD group (*p* < 0.05).

In contrast, entropy values were significantly lower in the IPD group (*p* < 0.05).

No statistically significant differences were observed between the groups for standard deviation, mean absolute deviation, median absolute deviation, maximum, variance, covariance, range, interquartile range, size-based histogram parameters (%L, %M, %U), kurtosis, skewness, smoothness, root-sum-of-squares, higher percentile values (95th, 97th, 99th), or uniformity (*p* > 0.05 for all) (Table 1).

### 3.3. GLRLM Analysis

In GLRLM analysis, the IPD group demonstrated a consistent pattern across multiple directions (0°, 45°, 90°, and 135°). Specifically, short run emphasis values were significantly lower, whereas long run emphasis values were significantly higher in the IPD group compared to controls (*p* < 0.05).

Furthermore, parameters reflecting gray-level distribution showed that low gray-level run emphasis and related metrics were significantly higher in the IPD group, whereas high gray-level run emphasis and associated parameters were significantly lower (*p* < 0.05).

Additional findings included significantly reduced run length nonuniformity and high gray-level nonuniformity, alongside increased long run low gray-level emphasis in the IPD group (*p* < 0.05).

These findings indicate a shift toward longer, lower gray-level runs in the IPD group, suggesting altered textural homogeneity of the optic nerve (Table 2 and Table 3).

#### GLSZM Analysis

GLSZM analysis demonstrated that the IPD group had significantly higher small zone emphasis and small zone low gray-level emphasis values, while large zone emphasis and large zone high gray-level emphasis values were significantly lower compared to controls (*p* < 0.05).

Additionally, gray-level variance and zone size variance were significantly increased in the IPD group (*p* < 0.05), indicating greater heterogeneity in gray-level distribution at the microstructural level.

Other GLSZM parameters, including low gray-level zone emphasis and zone percentage, were also significantly elevated in the IPD group (*p* < 0.05) (Table 3).

Following Benjamini–Hochberg false discovery rate (FDR) correction, only two marginally significant features (75th Percentile of Histogram and Run Percentage of GLRLM 45°) lost statistical significance, whereas all remaining significant radiomic features retained statistical significance after adjustment.

## 4. Discussion

In this study, we investigated microstructural changes in the optic nerve in patients with IPD using MRI-based tissue analyses. Our results show significant differences in various tissue parameters between IPD patients and healthy controls, suggesting subclinical involvement of the optic nerve in Parkinson’s disease.

Furthermore, these results provide novel evidence that the neurodegenerative process in IPD extends beyond the retina and encompasses the entire extraocular segment of the visual pathway. The most striking morphological finding in our study was a significant increase in ONSD in IPD patients. In the literature, ONSD is used as a non-invasive indicator in intracranial pressure assessment due to the relationship of the optic nerve sheath to the subarachnoid space [14,15].

However, in the context of neurodegeneration, this increase may reflect a different underlying mechanism. Recent evidence suggests that neuroinflammation within the optic nerve sheath and changes in cerebrospinal fluid dynamics may play a role in Parkinson’s disease [16]. Our observation of increased ONSD, along with decreased ROI area, points to a complex remodeling of the nerve and surrounding sheath; this may result from a loss of dopaminergic influence on vascular and fluid regulation in the orbital microenvironment. Dopamine is not only a neurotransmitter of the basal ganglia but is also found in the amacrine and interplexiform cells of the retina, where it modulates visual signal processing [17]. The reduction in retinal dopamine in IPD is known to impair the receptive field properties of ganglion cells, leading to decreased conduction capacity [18,19]. Our findings suggest that alterations associated with Parkinson’s disease may also be detectable within the extraocular segment of the optic nerve using quantitative MRI analysis.

Previous studies using OCT have consistently reported thinning of the retinal nerve fiber layer in IPD patients [20,21]. Our study demonstrates changes in the parenchymal signal intensity of the optic nerve. These changes may reflect axonal degeneration and demyelination within the optic nerve, similar to changes observed in the retina of Parkinsonian patients using OCT. Combining quantitative measurements of tissue analysis with brain MRI can increase sensitivity in detecting early neurodegenerative changes in IPD. These findings are also supported by diffusion tensor imaging studies demonstrating microstructural alterations of the visual pathways in newly diagnosed Parkinson’s disease [22]. Consistent with this evidence, our results suggest that MRI-based texture analysis may be sensitive to subtle optic nerve changes associated with Parkinsonian neurodegeneration.

In MRI texture analysis, higher intensity-based histogram parameters often correlate with subtle changes in water content or myelin integrity [8]. Our texture analysis revealed significantly higher mean, median, and percentile values of the histogram in the IPD group. Specifically, the decrease in Entropy in the IPD group is a critical finding. Entropy represents the degree of randomness or complexity in pixel distribution; Thus, lower entropy indicates a loss of structural heterogeneity [23]. This “simplification” of tissue architecture likely reflects the cumulative effect of axonal loss and the replacement of specialized neural structures with more uniform degenerative tissue [12].

Our findings should also be interpreted within the broader context of neurodegenerative diseases, in which visual pathway involvement is increasingly recognized as a component of multisystem neurodegeneration. Previous studies have demonstrated structural and microstructural alterations involving the retina and optic nerve in disorders such as Alzheimer’s disease, amyotrophic lateral sclerosis, and atypical Parkinsonian syndromes [10,24]. Although these diseases differ clinically and pathologically, they share several biological mechanisms including neuroaxonal degeneration, mitochondrial dysfunction, oxidative stress, chronic neuroinflammation, and abnormal protein accumulation. Such mechanisms may lead to disruption of tissue organization and progressive loss of structural complexity. In this context, the reduced entropy and altered gray-level distribution observed in our study may reflect not only local optic nerve involvement in IPD but also a more general pattern of neurodegenerative tissue remodeling. Nevertheless, because our study specifically evaluated patients with idiopathic Parkinson’s disease and did not include comparative neurodegenerative cohorts, these findings should not be interpreted as disease-specific imaging signatures. Comparative studies across different neurodegenerative conditions are warranted to determine whether optic nerve texture alterations represent a shared neurodegenerative phenotype or a distinct feature of Parkinsonian pathology.

GLRLM and GLSZM analyses demonstrated significant differences in gray-level nonuniformity and run-length features between IPD patients and controls. Specifically, the IPD group showed decreased short-run emphasis and increased long-run emphasis, supporting the presence of microstructural reorganization. In tissue analysis, similar grayscale intensities generally reflect a coarser and more homogeneous tissue and are often indicative of chronic pathological processes characterized by a loss of fine structural details. The loss of heterogeneous structures expected in normal tissue is usually explained in the literature by fibrotic remodeling or the dominance of the extracellular matrix as a result of a reduction in cellular elements. In such processes, the tissue acquires a biologically more “smooth” but functionally weaker structure [7,25]. These findings are consistent with prior histogram-based studies in optic neuritis, which have demonstrated that MRI-based texture features can detect subclinical optic nerve involvement even when conventional imaging appears unremarkable. In line with this, our results further suggest that such quantitative radiomic biomarkers are sensitive to subtle microstructural alterations, potentially reflecting early neurodegenerative processes in IPD.

The present findings may have important implications for future research. As the optic nerve represents an extension of the central nervous system, MRI-based texture analysis may provide a noninvasive approach for detecting subtle neurodegenerative changes beyond the retina. If validated in larger prospective cohorts, optic nerve texture features could potentially serve as adjunctive imaging biomarkers for the early detection of Parkinson’s disease. Furthermore, future studies are warranted to determine whether these quantitative parameters correlate with disease progression and clinical severity over time. Such investigations may help establish optic nerve texture analysis as a marker of neurodegeneration in Parkinson’s disease. Nevertheless, these findings should be considered exploratory. The excellent diagnostic performance observed in the present study, including the high AUC obtained for ONSD, may have been influenced by the retrospective single-center design and the relatively limited sample size, potentially leading to optimistic estimates of diagnostic accuracy. Therefore, independent external validation in larger, multicenter cohorts is essential before these imaging biomarkers can be considered reliable for routine clinical implementation.

Despite its significant findings, the present study has several limitations. First, its retrospective single-center design and the use of routine 1.5T MRI may have limited spatial resolution compared with dedicated high-resolution orbital MRI protocols. Second, although clear differences were observed between patients with IPD and healthy controls, comprehensive clinical data, including disease duration, disease severity, medication status, and visual symptoms, were not systematically available. Consequently, it was not possible to determine whether the observed MRI-based texture alterations were associated with disease severity, disease duration, treatment status, or visual dysfunction. Third, complementary assessments of the visual pathway, including optical coherence tomography (OCT), retinal nerve fiber layer measurements, visual evoked potentials, and detailed ophthalmological examinations, were unavailable, precluding direct correlation of MRI-derived texture features with functional or structural measures of visual pathway integrity. Finally, although ROIs were delineated by a single experienced radiologist using a predefined segmentation protocol, formal intraobserver and interobserver reproducibility analyses were not performed. In addition, because the ROI size was adjusted according to the anatomical diameter of the optic nerve to exclude the surrounding sheath and cerebrospinal fluid, the potential influence of ROI size on certain radiomics features cannot be completely excluded. Future prospective multicenter studies using high-resolution 3T MRI, multimodal ophthalmological assessments, standardized segmentation protocols, reproducibility analyses, and external validation cohorts are warranted to confirm the robustness and clinical applicability of these imaging biomarkers.

In conclusion, our findings suggest that MRI based texture analyses may detect subclinical microstructural alterations in the optic nerve of IPD patients. Integrating these quantitative imaging techniques with conventional clinical assessments may improve early diagnosis and monitoring of disease progression, contributing to a better understanding of Parkinson’s pathophysiology and the development of neuroprotective strategies.

## Figures and Tables

**Table 1:** Comparison of demographic characteristics and first-order histogram-based texture parameters between IPD patients and healthy controls.

		Control Group (n:37)				Case Group (n:37)				*p*	
		Mean ± sd			Median	Mean ± sd			Median		
***Histogram***											
Mean		196.3	±	47.2	187.1	260.1	±	119.5	208.7	***0.001***	^m^
Standard Deviation		40.5	±	14.1	39.0	58.0	±	54.4	35.2	0.618	^m^
Median		194.2	±	44.9	190.5	255.7	±	114.1	205.5	***0.001***	^m^
Mean Absolute Deviation		31.6	±	10.6	31.2	46.3	±	44.2	27.3	0.676	^m^
Median Absolute Deviation		24.1	±	9.5	23.0	37.1	±	39.0	22.8	0.544	^m^
Minimum		116.2	±	49.6	107.5	164.3	±	77.3	151.0	***0.000***	^m^
Maximum		300.7	±	94.4	278.0	394.5	±	240.5	283.5	0.223	^m^
Variance		1834	±	1340	1522	6288	±	13,567	1243	0.618	^m^
Covariance		1834	±	1340	1522	6288	±	13,567	1243	0.618	^m^
Range		184.5	±	78.5	168.0	230.2	±	206.7	145.5	0.279	^m^
Interquartile Range		50.6	±	18.5	49.3	79.7	±	82.8	47.0	0.700	^m^
Most Frequent Value		180.0	±	50.0	178.5	220.4	±	96.7	195.5	***0.012***	^m^
Size %L		14.9	±	4.5	15.9	15.8	±	5.1	16.1	0.210	^m^
Size %M		70.0	±	5.8	69.6	68.4	±	7.6	67.3	0.160	^t^
Size %U		15.1	±	3.3	15.0	15.8	±	4.5	15.8	0.329	^t^
Kurtosis		3.30	±	1.19	2.86	3.00	±	1.01	2.81	0.107	^m^
Skewness		0.23	±	0.76	0.24	0.32	±	0.68	0.27	0.486	^t^
Smoothness		0.001	±	0.001	0.001	0.001	±	0.001	0.001	0.618	^m^
Root-Mean-Square Level		200.6	±	48.0	191.5	267.8	±	128.0	212.3	***0.001***	^m^
Root-Sum-of-Squares Level		1721	±	447	1784	1811	±	723	1549	0.529	^m^
1st Percentile		117.9	±	48.7	109.3	164.6	±	77.1	151.0	***0.000***	^m^
3rd Percentile		126.0	±	46.9	115.5	170.5	±	76.2	158.1	***0.000***	^m^
5th Percentile		132.3	±	45.7	119.7	176.9	±	75.2	161.5	***0.000***	^m^
10th Percentile		146.0	±	44.2	133.9	189.7	±	77.3	172.3	***0.000***	^m^
25th Percentile		169.9	±	42.6	161.0	216.2	±	86.5	188.0	***0.000***	^m^
75th Percentile		220.5	±	50.9	214.1	296.0	±	149.8	230.0	***0.006***	^m^
90th Percentile		247.9	±	59.0	238.0	337.9	±	187.6	254.1	***0.020***	^m^
95th Percentile		267.0	±	68.1	252.4	364.6	±	213.3	271.1	0.051	^m^
97th Percentile		279.6	±	77.7	259.4	379.1	±	228.1	278.2	0.073	^m^
99th Percentile		296.7	±	90.4	274.7	393.8	±	240.6	283.3	0.164	^m^
Entropy		5.60	±	0.36	5.63	5.16	±	0.44	5.10	***0.000***	^t^
Uniformity		0.27	±	0.10	0.24	0.26	±	0.11	0.24	0.821	^m^

^m^ Mann–Whitney u test/^t^ Independent Samples *t* test.

**Table 2:** Quantitative GLRLM texture features of the optic nerve across different orientations (0° and 45°).

		Control Group (n: 37)				Case Group (n: 37)				*p*	
		Mean ± sd/n-%			Median	Mean ± sd/n-%			Median		
***GLRLM 000°***											
Short Run Emphasis		0.55	±	0.05	0.55	0.53	±	0.07	0.54	***0.048***	^m^
Long Run Emphasis		29.8	±	3.2	29.6	31.3	±	4.2	30.5	***0.048***	^m^
Gray-Level Nonuniformity		3.97	±	0.69	3.82	3.71	±	0.69	3.57	***0.014***	^m^
Run Length Nonuniformity		11.0	±	2.5	10.6	8.7	±	2.5	8.1	***0.000***	^m^
Run Percentage		0.27	±	0.02	0.28	0.26	±	0.03	0.27	0.056	^m^
Low Gray-Level Run Emphasis		0.35	±	0.06	0.34	0.39	±	0.06	0.39	***0.000***	^t^
High Gray-Level Run Emphasis		32.0	±	10.2	32.2	22.5	±	8.1	20.6	***0.000***	^m^
Short Run Low Gray-Level Emphasis		0.33	±	0.05	0.33	0.36	±	0.06	0.37	***0.011***	^t^
Short Run High Gray-Level Emphasis		2.66	±	0.96	2.61	1.95	±	0.89	1.77	***0.000***	^m^
Long Run Low Gray-Level Emphasis		1.32	±	0.57	1.21	2.12	±	1.26	1.71	***0.000***	^m^
Long Run High Gray-Level Emphasis		1908	±	626	1878	1336	±	489	1233	***0.000***	^m^
***GLRLM 045°***											
Short Run Emphasis		0.54	±	0.06	0.55	0.51	±	0.08	0.53	***0.040***	^m^
Long Run Emphasis		30.5	±	4.1	30.0	32.4	±	5.3	31.3	***0.040***	^m^
Gray-Level Nonuniformity		7.62	±	2.10	7.51	6.68	±	2.23	6.70	***0.009***	^t^
Run Length Nonuniformity		15.4	±	4.0	15.4	12.0	±	3.8	11.0	***0.000***	^m^
Run Percentage		0.39	±	0.04	0.39	0.37	±	0.05	0.38	***0.028***	^t^
Low Gray-Level Run Emphasis		0.47	±	0.05	0.47	0.49	±	0.06	0.50	***0.024***	^t^
High Gray-Level Run Emphasis		16.1	±	4.7	16.3	11.4	±	3.6	10.4	***0.000***	^m^
Short Run Low Gray-Level Emphasis		0.44	±	0.05	0.45	0.44	±	0.06	0.45	0.357	^m^
Short Run High Gray-Level Emphasis		1.28	±	0.35	1.31	0.95	±	0.32	0.94	***0.000***	^m^
Long Run Low Gray-Level Emphasis		2.65	±	1.23	2.26	3.93	±	1.50	3.63	***0.000***	^m^
Long Run High Gray-Level Emphasis		967	±	294	958	682	±	220	626	***0.000***	^m^

^m^ Mann–Whitney u test/^t^ Independent Samples *t* test.

**Table 3:** Comparative analysis of GLRLM (90° and 135°) and GLSZM (Gray-Level Size-Zone Matrix) features between IPD and control groups.

		Control Group (n: 37)				Case Group (n: 37)				*p*	
		Mean ± sd/n-%			Median	Mean ± sd/n-%			Median		
***GLRLM 090°***											
Short Run Emphasis		0.54	±	0.05	0.53	0.52	±	0.06	0.53	0.178	^m^
Long Run Emphasis		30.8	±	3.0	30.8	31.7	±	3.6	30.8	0.178	^m^
Gray-Level Nonuniformity		3.82	±	0.63	3.68	3.60	±	0.65	3.57	***0.040***	^m^
Run Length Nonuniformity		10.7	±	2.5	10.6	8.6	±	2.4	8.1	***0.000***	^m^
Run Percentage		0.27	±	0.03	0.28	0.26	±	0.03	0.27	0.201	^m^
Low Gray-Level Run Emphasis		0.34	±	0.04	0.33	0.38	±	0.05	0.37	***0.000***	^t^
High Gray-Level Run Emphasis		32.6	±	10.5	31.6	22.5	±	8.0	20.6	***0.000***	^m^
Short Run Low Gray-Level Emphasis		0.31	±	0.05	0.31	0.35	±	0.06	0.35	***0.000***	^t^
Short Run High Gray-Level Emphasis		2.82	±	1.01	2.60	2.00	±	0.96	1.85	***0.000***	^m^
Long Run Low Gray-Level Emphasis		1.86	±	1.40	1.21	2.40	±	1.79	1.79	***0.001***	^m^
Long Run High Gray-Level Emphasis		1941	±	651	1886	1334	±	483	1239	***0.000***	^m^
***GLRLM 135°***											
Short Run Emphasis		0.54	±	0.06	0.55	0.51	±	0.08	0.53	***0.039***	^m^
Long Run Emphasis		30.5	±	4.1	30.0	32.5	±	5.3	31.3	***0.039***	^m^
Gray-Level Nonuniformity		7.70	±	2.19	7.64	6.62	±	2.29	6.75	***0.004***	^t^
Run Length Nonuniformity		15.4	±	4.0	15.5	12.0	±	3.7	11.0	***0.000***	^m^
Run Percentage		0.39	±	0.04	0.39	0.37	±	0.05	0.38	***0.018***	^t^
Low Gray-Level Run Emphasis		0.47	±	0.05	0.48	0.49	±	0.07	0.49	0.067	^t^
High Gray-Level Run Emphasis		16.1	±	4.8	15.8	11.5	±	3.8	10.7	***0.000***	^m^
Short Run Low Gray-Level Emphasis		0.44	±	0.05	0.44	0.44	±	0.06	0.45	0.334	^m^
Short Run High Gray-Level Emphasis		1.28	±	0.36	1.31	0.97	±	0.33	0.94	***0.000***	^m^
Long Run Low Gray-Level Emphasis		2.72	±	1.17	2.49	3.80	±	1.70	3.59	***0.000***	^m^
Long Run High Gray-Level Emphasis		966	±	298	940	688	±	232	627	***0.000***	^m^
***GLSZM***											
Small Zone Emphasis		0.12	±	0.03	0.12	0.15	±	0.05	0.14	***0.000***	^t^
Large Zone Emphasis		35.9	±	11.1	34.3	25.8	±	10.3	23.5	***0.000***	^m^
Gray Level Nonuniformity		0.12	±	0.02	0.11	0.14	±	0.03	0.15	***0.000***	^m^
Zone Size Nonuniformity		0.11	±	0.02	0.11	0.14	±	0.03	0.14	***0.000***	^m^
Zone Percentage		0.19	±	0.03	0.19	0.23	±	0.04	0.23	***0.000***	^m^
Low Gray Level Zone Emphasis		0.12	±	0.04	0.12	0.17	±	0.06	0.15	***0.000***	^m^
High Gray Level Zone Emphasis		34.8	±	11.6	32.9	23.7	±	9.5	21.6	***0.000***	^m^
Small Zone Low Gray Level Emphasis		0.01	±	0.01	0.01	0.02	±	0.01	0.01	***0.000***	^m^
Small Zone High Gray Level Emphasis		3.64	±	0.95	3.75	3.06	±	0.94	2.97	***0.000***	^t^
Large Zone Low Gray Level Emphasis		3.89	±	1.11	3.75	3.81	±	1.31	3.53	0.462	^m^
Large Zone High Gray Level Emphasis		1350	±	862	1116	686	±	599	486	***0.000***	^m^
Gray Level Variance		0.002	±	0.001	0.002	0.003	±	0.002	0.003	***0.000***	^m^
Zone Size Variance		0.002	±	0.001	0.002	0.004	±	0.002	0.003	***0.000***	^m^

^m^ Mann–Whitney u test/^t^ Independent Samples *t* test.

## Data Availability

The data presented in this study are available on request from the corresponding author.

## Supplementary Materials

The following supporting information can be downloaded at: https://www.mdpi.com/article/10.3390/jcm15145388/s1, Table S1: Comparison Between the Right and Left Eyes Measurements.

## Abbreviations

IPD	Idiopathic Parkinson’s disease
OCT	Optical Coherence Tomography
GLRLM	Gray-level run-length matrix
GLSZM	Gray-level size-zone matrix
ONSD	Optic nerve sheath diameter
