# Supplementary material for: MRI-Based Texture Analysis of the Extraocular Optic Nerve in Idiopathic Parkinson’s Disease
Source: J Clin Med. 2026 Jul 9;15(14):5388. doi: 10.3390/jcm15145388 (PMC13410245; doi:10.3390/jcm15145388)
Supplement: Supplementary file 1 [file jcm-15-05388-s001.zip › jcm-4384126-supplementary.pdf]

Supplementary material Table S1: Comparision Between the Right and Left Eyes Measurements

	**Total, n (%)**	**Patient group, n (%)**	**Control group, n (%)**	**p**
	75 (100)	37 (49)	38 (51)	
Age, years, means ± SD	62.9 ± 11.3	63.5 ± 10.3	62.3 ± 12.3	0.650[a]
Gender				
Female	23 (31)	10 (27)	13 (34)	0.500[b]
Male	52 (69)	27 (73)	25 (66)	0.500[b]
Reft ONSD, means ± SD	0.41± 0.11	0.51± 0.07	0.32± 0.05	**<0.001**[a]
Left ONSD, means ± SD	0.42± 0.12	0.53± 0.07	0.32± 0.05	**<0.001**[a]

[a]Independent Samples t-test
[b]Chi-square test (data were shown as number and percentages)
